# Association between male absence and women’s reproductive autonomy in sub-Saharan Africa: A cross-sectional analysis of Demographic and Health Survey Data

**DOI:** 10.1371/journal.pgph.0007255

**Published:** 2026-09-15

**Authors:** Alex Bawuah, Ruth O. Ejiwale, Sylvester R. Okeke, Deborah O. Okeke-Obayemi, Sanni Yaya

**Affiliations:** 1 School of Global Studies, Faculty of Social Science, University of Sussex, Brighton, United Kingdom; 2 School of Health Sciences, Massey University, Auckland, New Zealand; 3 Centre for Social Research in Health, University of New South Wales, Sydney, Australia; 4 School of Social Sciences, University of New South Wales, Sydney, Australia; 5 School of Population Health, University of New South Wales, Sydney, Australia; 6 The George Institute for Global Health, Imperial College London, London, United Kingdom; University of Ghana, GHANA

## Abstract

Reproductive decision-making within partnered relationships in sub-Saharan Africa (SSA) is often shaped by patriarchal gender norms, with male partners commonly exerting substantial influence over family planning and contraception. These dynamics may shift when male partners migrate and are not physically present in the household. This study investigates the association between male absence and women’s reproductive autonomy in SSA, using nationally representative Demographic and Health Survey (DHS) data from 30 SSA countries. A total of 269,171 women aged 15–49 in unions (married or having a partner) and with at least one child were included in the study. We used a modified Poisson regression model to examine three outcomes - women’s desire for more children, use of modern contraceptives, and health autonomy - in relation to male absence as the primary explanatory variable, adjusting for sociodemographic and household characteristics. Overall, 29.35% of women reported using modern contraceptives, 61.04% desired more children, and 60.24% had autonomy in healthcare decision-making. Approximately 15.91% were not currently living with their husbands/partners. Women whose husbands/partners lived elsewhere had a lower likelihood of using modern contraceptives (PR = 0.80; 95% CI: 0.78 - 0.81) and a lower likelihood of desiring more children (PR = 0.95; 95% CI: 0.94 - 0.96), but higher likelihood of reporting health autonomy (PR = 1.10; 95% CI: 1.09 - 1.12). Furthermore, higher reproductive autonomy and contraceptive use were also associated with higher education levels, greater household wealth, urban residence, and employment. These findings highlight the complex ways in which male absence influence with women’s reproductive decision-making in SSA. Strengthening women’s reproductive autonomy will require addressing underlying structural inequalities, expanding educational and economic opportunities, and promoting awareness of reproductive rights. Targeted interventions that enhance equitable access to modern contraceptives are essential to reducing disparities within and across SSA countries.

## Introduction

Male absence from the household in sub-Saharan Africa (SSA), resulting from labour migration, the search for employment opportunities, or forced displacement due to armed conflict, has become an important demographic phenomenon in the region. This occurrence reorganises the structure of households in the region, impacting the pattern of reproductive practices and general reproductive health [1–3]. When men migrate, women tend to take up additional household burdens and responsibilities, including making health-related decisions [2,4]. While the absence of a male in SSA, known to be largely patriarchal [5,6], has the potential to be empowering for women to exert a higher level of reproductive autonomy, including birth control decisions, sociocultural norms, gendered power dynamics, and access to birth control, add complexities and may impact this relative freedom [7,8].

Reproductive autonomy, understood as the capacity to make a personal choice regarding reproductive health without being influenced and coerced [9], is a key aspect of gender equality and better outcomes of maternal health [10,11]. Nevertheless, in SSA, transferring family planning and contraception decision-making to male authority is common due to gender and sociocultural norms [12–15]. Research shows that in most of the rural and peri-urban settings in SSA, the accessibility and use of modern contraceptives by women continue to be highly dependent on the approval, presence and involvement of their male partners [16,17]. When men are non-present in the family setting as a result of labour migration, the absence of direct male supervision may lead to women taking control of their reproductive choices [18,19].

Male partner absence may influence women’s fertility preferences and contraceptive behaviour through several interconnected pathways. Although prolonged spousal separation may reduce coital frequency and, consequently, the immediate likelihood of conception, partner absence does not necessarily eliminate the need for contraception. Women may still choose to use contraception to postpone pregnancy until household circumstances become more stable, align childbearing with their reproductive intentions, or avoid unintended pregnancy when anticipating their partner’s return [20]. Furthermore, the temporary absence of a male partner may alter household decision-making dynamics, enabling women to make reproductive health decisions more independently in settings where men traditionally play a dominant role in family planning [21]. The economic and social changes associated with partner absence may also reshape fertility preferences, leading some women to delay or limit childbearing until household circumstances become more favourable [22].

Research on the association between male partner absence and women’s reproductive autonomy in sub-Saharan Africa (SSA) remains limited and inconclusive. Studies have reported differing associations between male partner absence and women’s reproductive autonomy, fertility preferences, and contraceptive use across SSA [20,21]. While some studies have found that women with absent partners exercise greater autonomy in reproductive decision-making [21], others suggest that this potential gain in autonomy is constrained by persistent sociocultural norms, unequal gender relations, economic dependence, and barriers to accessing family planning information and services [23,24]. Likewise, the association between partner absence and contraceptive use varies across settings and stages of migration, reflecting differences in the economic outcomes of migration, local gender norms, household arrangements, community contexts, and access to family planning services [20,25–27]. These inconsistencies underscore the need for further research to clarify how male partner absence influences women’s reproductive autonomy and contraceptive behaviour in SSA.

Thus, in an era of unprecedented migration [28,29], it is important to understand, more clearly and more deeply, the way male partner absence impacts reproductive autonomy among women. To contribute to this research effort, we drew from a large and high-quality data to understand the pattern of reproductive autonomy among women in SSA with migrating male partners.

We believe that research knowledge on the gendered association with male absence is vital in guiding specific policies that could improve reproductive autonomy and overall reproductive health and wellbeing of women. Through this study, we aim to inform and facilitate efforts to drive the realisation of Sustainable Development Goal 5 (gender equality) and Sustainable Development Goal 3 (health and well-being) by identifying contextual drivers and barriers to reproductive autonomy and how these barriers can be addressed in SSA.

## Methods

### Data source

We utilised the most recent Demographic and Health Surveys (DHS) dataset from 30 SSA countries, conducted between 2014 and 2024. The exact survey years for each country are provided in Table 1. Countries were included if they had a publicly available, nationally representative DHS dataset within this period that contained all key variables required for the analysis. Countries without a DHS survey in this timeframe or lacking essential variables were excluded. The countries included are Angola, Benin, Burkina Faso, Burundi, Cameroon, Chad, Côte d’Ivoire, Ethiopia, Gabon, Gambia, Ghana, Guinea, Kenya, Lesotho, Liberia, Madagascar, Malawi, Mali, Mauritania, Mozambique, Nigeria, Rwanda, Senegal, Sierra Leone, South Africa, Togo, Tanzania, Uganda, Zambia, and Zimbabwe. The DHS is a nationwide survey that is conducted in over 85 low- and middle-income countries worldwide and follows a consistent protocol and terminology across all countries [30]. It employs a structured questionnaire to gather information on various indicators of health, including maternal and child health, fertility, family planning utilisation, morbidity, and mortality [30]. The DHS follows a standardised methodology across countries and uses a two-stage stratified cluster sampling design. In the first stage, enumeration areas (EAs) are selected from the national sampling frame using probability proportional to size. In the second stage, a fixed number of households are randomly selected within each EA. This approach ensures that the survey is nationally representative of the population. Detailed information on the sampling and data collection methods can be found in the work of Aliaga and Ren [31].

**Table 1:** Prevalence of modern contraceptive use, desire for more children and health autonomy of women who are currently in a union across 30 SSA countries.

Country (year of survey)	Modern Contraceptive	More Children	Health Autonomy
	N (%)	N (%)	N (%)
Angola (2015–2016)	706 (9.26)	3,803 (52.51)	5,591 (73.30)
Burkina Faso (2021)	4,137 (34.74)	8,256 (72.07)	3,971 (33.34)
Benin (2017–2018)	1,385 (13.23)	6,515 (64.45)	4,941 (47.20)
Burundi (2016–2017)	2,146 (23.47)	4,591 (51.36)	6,667 (72.92)
Côte d’Ivoire (2021)	1,612 (18.15)	6,049 (72.14)	3,479 (39.18)
Cameroon (2018)	1,202 (17.54)	4,213 (65.35)	3,954 (57.71)
Ethiopia (2016)	2,653 (30.30)	5,254 (61.71)	7,140 (81.53)
Gabon (2019–2021)	659 (15.28)	2,377 (58.06)	3,190 (73.96)
Ghana (2022)	2,304 (28.20)	5,075 (65.27)	5,902 (72.23)
Gambia (2019–2020)	1,264 (17.81)	5,302 (76.53)	3,626 (51.10)
Guinea (2018)	680 (9.69)	4,583 (69.48)	2,725 (38.82)
Kenya (2022)	9,081 (52.29)	4,344 (49.52)	14,533 (83.68)
Liberia (2019–2020)	1,304 (29.52)	2,188 (51.16)	3,531 (79.92)
Lesotho (2023–2024)	2,085 (70.87)	941 (33.10)	2,756 (93.68)
Madagascar (2021)	4,733 (43.89)	5,859 (56.42)	9,358 (86.78)
Mali (2018)	1,224 (16.25)	5,125 (72.18)	1,582 (21.00)
Mauritania (2019–2021)	1,198 (13.97)	5,016 (60.81)	5,446 (63.50)
Malawi (2015–2016)	9,184 (61.49)	6,320 (48.21)	10,333 (69.19)
Mozambique (2022–2023)	2,392 (32.05)	3,732 (52.12)	5,555 (74.42)
Nigeria (2018)	3,361 (12.61)	16,960 (65.51)	11,900 (44.65)
Rwanda (2019–2020)	4,265 (61.48)	2,963 (45.04)	5,752 (82.92)
Sierra Leone (2019)	1,912 (20.83)	4,885 (55.53)	4,021 (43.82)
Senegal (2019)	2,509 (25.93)	6,888 (73.98)	3,332 (34.44)
Chad (2014–2015)	462 (3.84)	8,860 (79.70)	2,916 (24.26)
Togo (2013–2014)	1,076 (18.19)	3,323 (58.27)	2,519 (42.59)
Tanzania (2022)	2,681 (31.60)	5,101 (64.18)	6,478 (76.35)
Uganda (2016)	3,803 (35.86)	5,739 (56.99)	7,883 (74.34)
South Africa (2016)	1,463 (56.46)	759 (32.67)	2,418 (93.32)
Zambia (2018)	3,552 (49.01)	3,904 (55.80)	5,820 (80.30)
Zimbabwe (2015)	3,961 (70.68)	2,840 (51.59)	4,842 (86.40)
Total	78,994 (29.35)	151,765 (61.04)	162,161 (60.24)

We used the women’s dataset (IR file) from the DHS in the selected countries. The total sample consisted of 467,786 women aged 15–49, drawn from the pooled sample. This figure represents the unweighted sample size. For this study, we restricted the sample to women who were currently in a union (married or living with a partner) and who had at least one child. After applying these inclusion criteria and removing observations with missing data on the primary independent and dependent variables using listwise deletion, the final analytic sample consisted of 269,171 women, reflecting the unweighted analytic sample**.**

### Variables

The study examined three reproductive health-related outcomes: (i) modern contraceptive use, (ii) fertility desire, and (iii) women’s participation in healthcare decision-making. These indicators were selected because they reflect important dimensions of reproductive behaviour, preferences, and agency that are commonly examined in Demographic and Health Survey (DHS)-based reproductive health research. In particular, women’s participation in healthcare decision-making was included as a proxy dimension of women’s agency because it reflects women’s involvement in decisions regarding their own healthcare. However, we recognise that reproductive autonomy is multidimensional and cannot be fully captured through any single indicator or behavioural outcome.

Modern contraceptive use was assessed using the DHS question: “Which contraceptive method are you using?” (DHS variable v313). The responses include: (i) no method, (ii) folkloric method, (iii) traditional method, and (iv) modern method. For this study, the variable was recoded into a binary outcome: 1 = using any modern method, and 0 = not using a modern method (i.e., no method, folkloric method, and traditional method).

The desire for more children was based on the DHS question: “After the child you are expecting now, would you like to have another child, or would you prefer not to have any more children?” (DHS variable v602). The response categories include: (i) have another child, (ii) undecided, (iii) no more, (iv) sterilized, and (v) declared infecund. We created a binary variable coded as 1 = desire to have another child, and 0 = does not desire another child (a combination of “undecided” and “no more”). Respondents who reported being sterilized or infecund were excluded from this analysis.

Autonomy in health decisions was measured using the DHS question: “Who usually makes decisions about health care for yourself: you, your (husband/partner), you and your (husband/partner) jointly, or someone else?” (DHS variable v743a). Response options include: (i) respondent alone, (ii) respondent and husband/partner, (iii) husband/partner alone, (iv) someone else, and (v) other. For this study, women are considered to have health autonomy if they make healthcare decisions either alone or jointly with their husbands/partners. Accordingly, the variable was recoded into a binary indicator where 1 = respondent has health autonomy (alone or jointly with husband/partner) and 0 = respondent does not have health autonomy (health decisions are made by husband/partner alone, someone else, or other).

The main independent variable was male partner absence, measured using the DHS variable on the current residence status of the respondent’s husband/partner. Respondents were asked whether they were currently living with their husband/partner. Responses were coded as 1 = living with husband/partner and 0 = husband/partner staying elsewhere. Respondents whose husbands/partners were reported as “staying elsewhere” were classified as experiencing male partner absence. However, the DHS does not provide information on the reason, duration, or nature of the partner’s absence. Consequently, this measure may capture different forms of non-co-residence, including labour-related mobility, temporary absence, duolocal residence arrangements, or marital separation. The following variables are included as covariates: age (15–24, 25–34, 35–49), education (no education, primary, secondary, higher), wealth (poorest, poorer, middle, richer, richest), residence (urban, rural), number of living children (1–2, 3–5, ≥ 6), frequency of reading newspapers (not at all, less than once a week, at least once a week, almost every day), frequency of listening to radio (not at all, less than once a week, at least once a week, almost every day), frequency of watching television (not at all, less than once a week, at least once a week, almost every day) and employment status (employed, unemployed). These variables were selected based on their significant relationship with the outcome variables in the literature [26,32–34] as well as their availability in the DHS dataset.

### Data analysis

The data was analysed with STATA version 18. Descriptive statistics were used to describe the sample as well as the prevalence of modern contraceptive use, desire for more children and health autonomy across the 30 SSA countries.

Given that the study outcomes were binary, we estimated associations using modified Poisson regression models with a log link function and robust standard errors. This approach was selected because it directly estimates prevalence ratios, which are more interpretable than odds ratios in cross-sectional studies. In addition, the outcomes examined in this study were relatively common (prevalence ≥10%), and odds ratios from logistic regression may overestimate the magnitude of association under such circumstances. Modified Poisson regression therefore provides valid and easily interpretable estimates of association for common binary outcomes. [35–37]. We first conducted univariate analyses to estimate crude risk ratios (results presented in S1 Appendix), followed by multivariable modified Poisson regression models to estimate adjusted risk ratios.

The DHS survey design - sampling weights (v005), primary sampling units (v021), and stratification (v022) - was incorporated into all regression analyses. Following DHS analytical guidelines [38], we generated a normalized weight variable (gen wt = v005/1,000,000) and set the survey design using: svyset [pw = wt], psu(v021) strata(v022). All analyses were then conducted using the survey (‘svy’) commands to account for the complex sampling design. All analyses were conducted using pooled data from the 30 included countries. Country-level fixed effects were included to account for between-country differences in demographic, socioeconomic, and health system characteristics.

Prior to conducting the regression analysis, we performed a diagnostic test for collinearity between the independent variables by examining the variance inflation factor (VIF) and found no signs of multicollinearity. The results are provided in S2 Appendix.

### Ethical consideration

We used a secondary dataset that is freely available to the public from the DHS Programme, therefore no ethical approval was requested. The dataset is anonymised and more details regarding its ethical standards are available at http://goo.gl/ny8T6X.

## Results

### Prevalence of modern contraceptive use, desire for more children and health autonomy of women who are currently in a union across 30 SSA countries

Table 1 reports the prevalence of modern contraceptive use, desire for more children and health autonomy of women (15–49 years) who are currently in a union across 30 SSA countries. The results show that Chad has the lowest prevalence of women using modern contraceptives (3.84%), whereas Zimbabwe has the highest prevalence of women using modern contraceptives (70.68%). Furthermore, South Africa has the lowest prevalence of women who desire more children (32.67%), whereas Chad has the highest prevalence of women who desire more children (79.70%). Mali has the lowest prevalence of women with health autonomy (21%), whereas South Africa has the highest prevalence of women with health autonomy (93.32%).

### Descriptive summary of the sample based on key outcome variables

**Fig 1:**
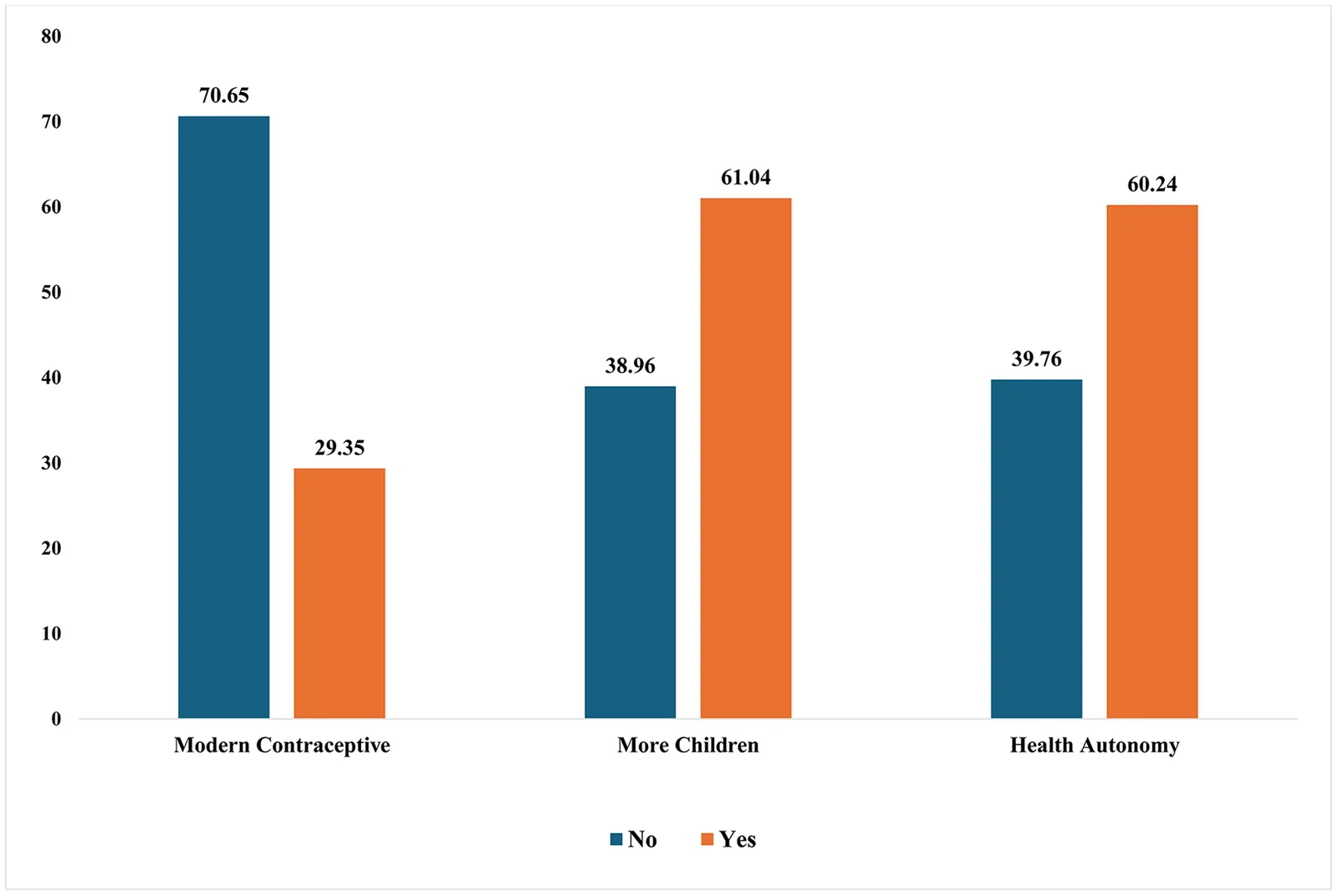
Distribution of the three outcome variables from the study sample.

Fig 1 shows the distribution of the study sample on the three outcome variables. It shows that 29.35% of the study sample use modern contraceptives, 61.04% desire more children, and 60.24% have health autonomy.

Table 2 presents the results of the descriptive summary of the sample. It shows that 15.91% of the women are not currently living with their husbands/partners. Furthermore, a higher proportion of the women (42.85%) were between 25 and 34 years, have no education (40.73%), are in the poorest wealth category (22.41%), live in rural areas (66.40%), have between 3–5 children (44.89%), currently employed (63.11%), do not read newspapers (87.79%), do not listen to the radio (46.22%), and do not watch television (59.01%).

**Table 2:** Descriptive summary of the sample.

	Total Sample n = 269,171	Modern Contraceptive n = 78,994	More Children n = 151,765	Health Autonomy n = 162,161
	N (%)	N (%)	N (%)	N (%)
**Spousal Residence Status**				
Living with husband/partner	226,345 (84.09)	68,404 (86.59)	126,849 (83.58)	133,974 (82.62)
Husband/partner lives elsewhere	42,826 (15.91)	10,590 (13.41)	24,916 (16.42)	28,187 (17.38)
**Age**				
15-24	53,524 (19.88)	15,350 (19.43)	45,583 (30.04)	28,237 (17.41)
25-34	108,364 (40.26)	33,872 (42.88)	74,188 (48.88)	64,526 (39.79)
35-49	107,283 (39.86)	29,772 (37.69)	31,994 (21.08)	69,398 (42.80)
**Education level**				
No education	109,642 (40.73)	17,419 (22.05)	63,725 (41.99)	49,586 (30.58)
Primary	84,839 (31.52)	31,635 (40.05)	43,198 (28.46)	57,700 (35.58)
Secondary	62,603 (23.26)	24,705 (31.27)	38,283 (25.23)	44,736 (27.59)
Higher	12,087 (4.49)	5,235 (6.63)	6,559 (4.32)	10,139 (6.25)
**Wealth**				
Poorest	60,323 (22.41)	12,612 (15.97)	35,428 (23.34)	32,475 (20.03)
Poorer	54,993 (20.43)	14,509 (18.37)	31,515 (20.77)	31,098 (19.18)
Middle	54,216 (20.14)	15,970 (20.22)	30,278 (19.95)	31,960 (19.71)
Richer	51,476 (19.12)	17,381 (22.00)	28,442 (18.74)	32,452 (20.01)
Richest	48,163 (17.89)	18,522 (23.45)	26,102 (17.20)	34,176 (21.08)
**Residence**				
Urban	90,444 (33.60)	29,018 (36.73)	50,477 (33.26)	59,208 (36.51)
Rural	178,727 (66.40)	49,976 (63.27)	101,288 (66.74)	102,953 (63.49)
**Number of Children**				
1-2 Children	99,849 (37.10)	29,742 (37.65)	79,050 (52.09)	60,768 (37.47)
3-5 Children	120,819 (44.89)	37,874 (47.95)	60,630 (39.95)	73,365 (45.24)
≥6 Children	48,503 (18.02)	11,378 (14.40)	12,085 (7.96)	28,028 (17.28)
**Frequency of reading newspaper**				
Not at all	236,295 (87.79)	64,552 (81.72)	134,191 (88.42)	137,040 (84.51)
Less than once a week	20,123 (7.48)	8,812 (11.16)	11,009 (7.25)	15,125 (9.33)
At least once a week	12,365 (4.59)	5,472 (6.93)	6,350 (4.18)	9,687 (5.97)
Almost every day	388 (0.14)	158 (0.20)	215 (0.14)	309 (0.19)
**Frequency of listening to radio**				
Not at all	124,423 (46.22)	29,290 (37.08)	72,063 (47.48)	68,922 (42.50)
Less than once a week	53,142 (19.74)	16,102 (20.38)	31,176 (20.54)	32,683 (20.15)
At least once a week	87,139 (32.37)	32,292 (40.88)	46,279 (30.49)	57,554 (35.49)
Almost every day	4,467 (1.66)	1,310 (1.66)	2,247 (1.48)	3,002 (1.85)
**Frequency of watching television**				
Not at all	158,840 (59.01)	41,463 (52.49)	89,094 (58.71)	90,381 (55.74)
Less than once a week	34,196 (12.70)	10,388 (13.15)	20,040 (13.20)	20,816 (12.84)
At least once a week	70,893 (26.34)	25,421 (32.18)	39,759 (26.20)	47,155 (29.08)
Almost every day	5,242 (1.95)	1,722 (2.18)	2,872 (1.89)	3,809 (2.35)
**Currently working**				
No	99,309 (36.89)	25,955 (32.86)	60,145 (39.63)	52,239 (32.21)
Yes	169,862 (63.11)	53,039 (67.14)	91,620 (60.37)	109,922 (67.79)

For women whose husbands/partners live elsewhere, 13.41% use modern contraceptives, 16.42% desire to have more children, and 17.38% have health autonomy. A higher proportion of the women who use modern contraceptives (40.05%) and have health autonomy (35.58%) have primary education, whereas those who desire more children have no education (41.99%). Moreover, a higher proportion of the women who use modern contraceptives (23.45%) and have health autonomy (21.08%) are in the richest wealth category, whereas those who desire more children are in the poorest wealth category (23.34%). Additionally, a higher proportion of the women who use modern contraceptives (47.95%) and have health autonomy (45.24%) have between three and five children, whereas those who desire more children have between one and two children (52.09%).

### Relationship between male absence and women’s desire for more children, use of modern contraceptives, and health autonomy

Table 3 presents findings from the multivariable modified Poisson regression models on the relationship between male absence and women’s desire for more children, use of modern contraceptives, and health autonomy.

**Table 3:** A modified Poisson regression model examining the relationship between male absence and women’s desire for more children, use of modern contraceptives, and health autonomy.

	Modern Contraceptive	More Children	Health Autonomy
Variables	PR (95%CI)	PR (95%CI)	PR (95%CI)
**Spousal Residence Status**			
Living with husband/partner	Yes	Yes	Yes
Husband/partner lives elsewhere	0.80 (0.78 - 0.81)***	0.95 (0.94 - 0.96)***	1.10 (1.09 - 1.12)***
**Age**			
15-24	Yes	Yes	Yes
25-34	0.93 (0.92 - 0.95)***	0.98 (0.98 - 0.99)***	1.10 (1.09 - 1.11)***
35-49	0.78 (0.76 - 0.80)***	0.57 (0.57 - 0.58)***	1.21 (1.19 - 1.22)***
**Education level**			
No education	Yes	Yes	Yes
Primary	1.46 (1.43 - 1.50)***	0.94 (0.93 - 0.95)***	1.15 (1.14 - 1.17)***
Secondary	1.63 (1.58 - 1.68)***	0.97 (0.96 - 0.98)***	1.25 (1.23 - 1.27)***
Higher	1.61 (1.55 - 1.68)***	1.01 (0.98 - 1.03)	1.31 (1.28 - 1.34)***
**Wealth**			
Poorest	Yes	Yes	Yes
Poorer	1.15 (1.12 - 1.18)***	0.97 (0.96 - 0.98)***	1.02 (1.00 - 1.04)*
Middle	1.22 (1.19 - 1.26)***	0.96 (0.95 - 0.97)***	1.03 (1.01 - 1.05)***
Richer	1.26 (1.22 - 1.29)***	0.94 (0.93 - 0.95)***	1.05 (1.03 - 1.07)***
Richest	1.22 (1.18 - 1.26)***	0.92 (0.90 - 0.94)***	1.06 (1.04 - 1.08)***
**Residence**			
Urban	Yes	Yes	Yes
Rural	0.95 (0.93 - 0.97)***	1.04 (1.03 - 1.05)***	0.96 (0.95 - 0.98)***
**Number of Children**			
1-2 Children	Yes	Yes	Yes
3-5 Children	1.30 (1.27 - 1.32)***	0.71 (0.70 - 0.72)***	1.00 (0.99 - 1.01)
≥6 Children	1.32 (1.28 - 1.35)***	0.42 (0.41 - 0.43)***	0.99 (0.98 - 1.00)
**Frequency of reading newspaper**			
Not at all	Yes	Yes	Yes
Less than once a week	1.01 (0.99 - 1.03)	0.99 (0.97 - 1.00)	1.00 (0.99 - 1.02)
At least once a week	1.01 (0.98 - 1.04)	0.99 (0.97 - 1.01)	0.99 (0.98 - 1.00)
Almost every day	1.39 (1.16 - 1.68)***	1.06 (0.97 - 1.16)	0.97 (0.91 - 1.02)
**Frequency of listening to radio**			
Not at all	Yes	Yes	Yes
Less than once a week	1.08 (1.06 - 1.11)***	1.02 (1.01 - 1.03)***	1.06 (1.05 - 1.07)***
At least once a week	1.09 (1.07 - 1.11)***	1.02 (1.01 - 1.03)**	1.04 (1.03 - 1.05)***
Almost every day	1.09 (1.02 - 1.16)*	0.96 (0.92 - 0.99)*	1.01 (0.99 - 1.04)
**Frequency of watching television**			
Not at all	Yes	Yes	Yes
Less than once a week	1.08 (1.05 - 1.11)***	0.99 (0.98 - 1.00)	1.04 (1.03 - 1.06)***
At least once a week	1.11 (1.09 - 1.14)***	0.98 (0.97 - 0.99)**	1.04 (1.03 - 1.06)***
Almost every day	1.30 (1.21 - 1.38)***	1.01 (0.97 - 1.05)	0.97 (0.94 - 1.00)
**Currently working**			
No	Yes	Yes	Yes
Yes	1.18 (1.16 - 1.20)***	1.02 (1.01 - 1.02)***	1.21 (1.20 - 1.23)***
**Country fixed effect**	Yes	Yes	Yes
**Constant**	0.05 (0.05 - 0.06)***	0.82 (0.79 - 0.85)***	0.49 (0.47 - 0.51)***
**Observations**	269,171	248,620	269,171

PR = Prevalence Ratio; 95% confidence interval (CI) in parentheses; Ref = Reference group.

*** p < 0.001, ** p < 0.01, * p < 0.05.

The results showed that women whose husbands/partners lived elsewhere were 20% less likely to use modern contraceptives compared to those who lived with their husbands/partners (PR = 0.80; 95% CI: 0.78 - 0.81). Similarly, they had a 5% lower likelihood of desiring more children (PR = 0.95; 95% CI: 0.94 - 0.96). However, they were 10% more likely to have autonomy in making healthcare decisions compared to women living with their husbands/partners (PR = 1.10; 95% CI: 1.09 - 1.12).

Women aged 35–49 years had a 22% lower likelihood of using modern contraceptives (PR = 0.78; 95% CI: 0.76 - 0.80) and were 43% less likely to desire more children (PR = 0.57; 95% CI: 0.57 - 0.58) compared to those aged 15–24 years. Conversely, they had a 21% higher likelihood of having health autonomy than women aged 15–24 years (PR = 1.21; 95% CI: 1.19 - 1.22).

Furthermore, women with a secondary level of education were 63% more likely to use modern contraceptives (PR = 1.63; 95% CI: 1.58 - 1.68) and had a 25% higher likelihood of having health autonomy compared to those with no education (PR = 1.25; 95% CI: 1.23 - 1.27). However, they were 3% less likely to desire more children than women with no education (PR = 0.97; 95% CI: 0.96 - 0.98).

Women in the richest wealth category had a 22% higher likelihood of using modern contraceptives (PR = 1.22; 95% CI: 1.18 - 1.26) and were 6% more likely to have health autonomy compared to those in the poorest category (PR = 1.06; 95% CI: 1.04 - 1.08). However, they had an 8% lower likelihood of desiring more children (PR = 0.92; 95% CI: 0.90 - 0.94).

Women residing in rural areas were 5% less likely to use modern contraceptives (PR = 0.95; 95% CI: 0.93 - 0.97) and had a 4% lower likelihood of having health autonomy compared to those living in urban areas (PR = 0.96; 95% CI: 0.95 - 0.98). In contrast, they were 4% more likely to desire more children than urban residents (PR = 1.04; 95% CI: 1.03 - 1.05).

Women with six or more children had a 32% higher likelihood of using modern contraceptives (PR = 1.32; 95% CI: 1.28 - 1.35) compared to those with one or two children. However, they were 58% less likely to desire more children (PR = 0.42; 95% CI: 0.41 - 0.43) and had no statistically significant difference in likelihood of having health autonomy (PR = 0.99; 95% CI: 0.98 - 1.00).

The results further revealed that women who are currently employed were 18% more likely to use modern contraceptives (PR = 1.18; 95% CI: 1.16 - 1.20), 2% more likely to desire more children (PR = 1.02; 95% CI: 1.01 - 1.02), and 21% more likely to have health autonomy (PR = 1.21; 95% CI: 1.20 - 1.23) compared to the unemployed

## Discussion

We found that about a third of women in union use modern family planning, about three in five women desire more children (61.04%), and have health autonomy (60.24%). The relatively low level of women using modern contraceptives found in our study compares with the low level of contraceptive coverage and use in SSA [23,24,26,27,39]. It is concerning to see a high level of within and between countries disparity in the use of modern contraceptives in our sample, as reported in previous studies [23,26,39]. Chad’s significantly low level of modern contraceptive use may further bring to the fore, the place of fragility and political instability on health and wellbeing. Instability could impact the availability, accessibility and effectiveness of health services, including maternal and reproductive healthcare [23,25,26]. Moreover, it is interesting to note that Chad, with the lowest level of modern contraceptive use, also reported the strongest desire for more children. Aside from the impact of the socio-political environment and healthcare performance, it is likely that this desire for more children may be impacting utilisation of modern contraceptive use among women in Chad. Previous studies in SSA have shown that the fear of adverse impact on fertility acts as a barrier to women’s use of modern contraceptives [40,41]. It is important to stress that health education and promotion efforts may be impactful in empowering women to understand that the use of modern contraceptives does not foreclose or reduce the chances of having more children.

Overall, our investigation showed that the impact of male absence on women’s reproductive autonomy is somewhat complex. We found that women with partners residing in other places are much less prone to using modern contraceptives, intend to have more children and are more independent in making health-related decisions. This complexity represents the two-sided effect of male absence, which, on the one hand, may improve reproductive autonomy [7,18,22] and, on the other hand, may also decrease communication and partner support in women’s use of family planning [2,4,8]. Notably, low utilisation of modern contraceptives among women with migrating male partners may be attributable to the low possibility of falling pregnant since the male partner is away. While this may be plausible, there is also a possibility of the migrating partner paying an unscheduled visit. Such unscheduled and/or unplanned visits may result in unplanned pregnancy, hence the need for women with migrating partners to consider using modern contraceptives if there is a need for birth control. The absence of a male partner may not be a sufficient protective factor against unplanned pregnancy.

Our analysis revealed how a number of factors account for differences in reproductive health outcomes and reproductive autonomy among the women in our sample. In line with evidence from previous studies [26,39,42], education remains a significant and strong predictor of contraceptive use and reproductive autonomy among our sample. We found that women in our sample who attained secondary education or above were more likely to also report higher modern contraceptive use, higher reproductive autonomy and lower desire for more children. This finding could be attributable to the fact that women with formal education are more likely to possess higher reproductive health literacy as well as more likely than their less or non-formal educated peers to possess the required confidence to negotiate or autonomously make contraceptive use decisions [24,43,44]. The empowering impact of education on reproductive health decisions and outcomes are even stronger when women and their male partners both possess a considerably high level of formal education. This exposure to formal education could contribute to male partners’ willingness to support their female partners’ utilisation of modern contraceptives and overall reproductive health [17,45,46].

Aside from reproductive health literacy, formal education may also improve reproductive health autonomy, decisions and outcomes for women because of higher economic power. As such, in line with previous studies [47,48], we found that women in our sample in the highest economic quintile also have greater chances of reporting higher reproductive autonomy, lower desire for more children and higher contraceptive use. Women with higher economic status, when compared to their peers living in poverty, will have greater access to media exposure and health services [25–27]. Just as we found among our sample, and in line with previous studies [14–16], the media has the tendency of normalising contraceptive use, thereby resulting in behavioural change in people with media exposure to access and utilise modern contraceptive use. As a result, economic power provides a safety net by exposing these women to media contents that keep them informed of reproductive health information and their reproductive health rights, including the right to access and utilise modern contraceptives. Against this backdrop, it is important to consider media campaigns targeting less or non-formally educated women of childbearing age to improve their knowledge and social competence to exercise their reproductive health rights and autonomy.

We also found a complex relationship between age and reproductive health outcomes. For instance, we found that older women (35–49 years) were more likely to report higher reproductive health autonomy than younger women (15–24 years), but lower contraceptive use. This implies that even though older women possess higher reproductive autonomy, this autonomy is not translated into actions to result in high contraceptive use. The complexity of this relationship may also be attributable to the fact that these older women also have a lower desire for more children, probably because they may have already given birth to their desired number of children. However, considering that these older women are still within the reproductive age group, it is expected that they should report higher contraceptive use to reduce or outrightly forestall the possibility of falling pregnant. Our results support previous studies [49,50] that have documented lower contraceptive use among older reproductive age women. This has implications for public health education and promotion to improve modern contraceptives use among older women who are still within the reproductive age group. Our results also have implications for efforts and interventions targeting younger women of reproductive age to understand and exercise their reproductive health and rights.

In line with previous studies [51,52], we found that women in employment are more likely to report higher reproductive autonomy and contraceptive use than those not in employment. Being in employment could also be an indicator of possessing formal education, and the world of work provides additional exposure that could further reinforce the benefit of education on reproductive health outcomes. Also, being in employment could also indicate a relatively higher economic power. Women in employment may have a higher level of economic independence than their peers who are not employed. Women’s economic independence has been reported to improve women’s economic autonomy and negotiation capacity [4,50]. This may explain the greater reproductive autonomy that women in our sample who are employed reported. As such, girls’ and women’s education could play a significant role in social stability as it does not only address knowledge gaps, but also economic empowerment and reproductive health and rights.

### Recommendations

The findings of this study underscore the need for comprehensive strategies to strengthen women’s reproductive autonomy in sub-Saharan Africa, particularly in contexts shaped by male absence. Policies should prioritise improving equitable access to modern contraceptives (particularly in rural and underserved areas) through strengthened supply chains and community-based services. Expanding girls’ and women’s education remains essential, as higher schooling strongly predicts autonomy and contraceptive use. Economic empowerment initiatives, including employment opportunities and social protection, can further enhance women’s decision-making power. Targeted health communication campaigns are also needed to address misconceptions about modern contraceptives and promote informed reproductive decision-making. Finally, interventions should consider the unique circumstances of women whose partners migrate, including counselling and support to help them plan and exercise their reproductive choices autonomously.

### Strengths and limitations

Our study uses a large sample and robust and high-quality data. The DHS dataset is globally recognised for its high-quality data collected through rigorous and standardized protocols. Drawing from the DHS data set implies that our study results and conclusion are highly valid and reliable because of the large and representative sample as well as the sophisticated statistical techniques used for analysis. The advanced statistical techniques we use in analysing our data further strengthen the reliability and validity of our results.

However, our results need to be understood within some caveats. The DHS protocols involve self-reporting with elements of social desirability bias and underreporting of behaviours that may be considered as being against acceptable social norms, such as contraceptive use without the consent/approval of male partners. Aside from social desirability bias, there are also concerns relating to recall bias that could impact the accuracy of data. Finally, reproductive autonomy in this study is operationalised as reproductive-health decision-making, which may not fully capture the multidimensional nature of reproductive autonomy.

## Conclusion

Based on the findings of our study, it is important to expedite actions and interventions to address disparities between and within countries in the utilisation of modern contraceptives among women of reproductive age in sub-Saharan Africa. A key strategy to addressing this disparity would include education and empowerment for women to understand and exercise their reproductive health rights and autonomy. We found that countries with low levels of reproductive autonomy are also more likely to report low modern contraceptive use. It is also important to address structural inequalities by ensuring equitable access to education, economic opportunities and media exposure.

## Supporting information

S1 Appendix Results from univariate regression analysis. (DOCX)

S2 Appendix Collinearity diagnostics. (DOCX)
