# Supplementary material for: Association between male absence and women’s reproductive autonomy in sub-Saharan Africa: A cross-sectional analysis of Demographic and Health Survey Data
Source: PLOS Glob Public Health. 2026 Sep 15;6(9):e0007255. doi: 10.1371/journal.pgph.0007255 (PMC13577400; doi:10.1371/journal.pgph.0007255)
Supplement: Supplementary file 1 [file pgph.0007255.s001.docx]

**Results from univariate regression analysis**

|  | **Modern Contraceptive** | **More Children** | **Health Autonomy** |  |  |
| --- | --- | --- | --- | --- | --- |
| **Variables** | **PR (95%CI)** | **PR (95%CI)** | **PR (95%CI)** |  |  |
| **Spousal Residence Status** |  |  |  |  |  |
| Living with husband/partner | Ref | Ref | Ref |  |  |
| Husband/partner lives elsewhere | 0.82 (0.80 - 0.85)*** | 1.03 (1.02 - 1.04)*** | 1.11 (1.09 - 1.12)*** |  |  |
| **Age** |  |  |  |  |  |
| 15-24 | Ref | Ref | Ref |  |  |
| 25-34 | 1.10 (1.08 - 1.13)*** | 0.82 (0.81 - 0.82)*** | 1.14 (1.13 - 1.16)*** |  |  |
| 35-49 | 0.97 (0.95 - 0.99)* | 0.38 (0.38 - 0.39)*** | 1.24 (1.23 - 1.26)*** |  |  |
| **Education level** |  |  |  |  |  |
| No education | Ref | Ref | Ref |  |  |
| Primary | 2.23 (2.17 - 2.29)*** | 0.91 (0.89 - 0.92)*** | 1.51 (1.49 - 1.54)*** |  |  |
| Secondary | 2.36 (2.29 - 2.43)*** | 1.06 (1.05 - 1.07)*** | 1.60 (1.57 - 1.63)*** |  |  |
| Higher | 2.54 (2.44 - 2.65)*** | 1.03 (1.00 - 1.05)* | 1.87 (1.84 - 1.91)*** |  |  |
| **Wealth** |  |  |  |  |  |
| Poorest | Ref | Ref | Ref |  |  |
| Poorer | 1.24 (1.20 - 1.28)*** | 0.98 (0.97 - 1.00)** | 1.06 (1.04 - 1.08)*** |  |  |
| Middle | 1.40 (1.35 - 1.45)*** | 0.96 (0.94 - 0.97)*** | 1.13 (1.10 - 1.15)*** |  |  |
| Richer | 1.57 (1.52 - 1.63)*** | 0.95 (0.94 - 0.97)*** | 1.22 (1.19 - 1.24)*** |  |  |
| Richest | 1.72 (1.66 - 1.79)*** | 0.93 (0.92 - 0.95)*** | 1.35 (1.32 - 1.38)*** |  |  |
| **Residence** |  |  |  |  |  |
| Urban | Ref | Ref | Ref |  |  |
| Rural | 0.91 (0.88 - 0.94)*** | 1.01 (1.00 - 1.03) | 0.87 (0.86 - 0.89)*** |  |  |
| **Number of Children** |  |  |  |  |  |
| 1-2 Children | Ref | Ref | Ref |  |  |
| 3-5 Children | 1.06 (1.04 - 1.07)*** | 0.64 (0.63 - 0.64)*** | 1.00 (0.99 - 1.01) |  |  |
| ≥6 Children | 0.79 (0.77 - 0.81)*** | 0.32 (0.31 - 0.33)*** | 0.94 (0.93 - 0.95)*** |  |  |
| **Frequency of reading newspaper** |  |  |  |  |  |
| Not at all | Ref | Ref | Ref |  |  |
| Less than once a week | 1.53 (1.49 - 1.57)*** | 0.98 (0.97 - 1.00)* | 1.30 (1.28 - 1.32)*** |  |  |
| At least once a week | 1.56 (1.51 - 1.61)*** | 0.93 (0.91 - 0.96)*** | 1.36 (1.34 - 1.38)*** |  |  |
| Almost every day | 1.54 (1.32 - 1.80)*** | 0.97 (0.87 - 1.09) | 1.39 (1.32 - 1.47)*** |  |  |
| **Frequency of listening to radio** |  |  |  |  |  |
| Not at all | Ref | Ref | Ref |  |  |
| Less than once a week | 1.26 (1.23 - 1.29)*** | 1.02 (1.01 - 1.03)** | 1.12 (1.10 - 1.14)*** |  |  |
| At least once a week | 1.56 (1.52 - 1.59)*** | 0.96 (0.94 - 0.97)*** | 1.21 (1.19 - 1.23)*** |  |  |
| Almost every day | 1.21 (1.13 - 1.30)*** | 0.85 (0.82 - 0.88)*** | 1.24 (1.20 - 1.29)*** |  |  |
| **Frequency of watching television** |  |  |  |  |  |
| Not at all | Ref | Ref | Ref |  |  |
| Less than once a week | 1.15 (1.12 - 1.18)*** | 1.04 (1.02 - 1.05)*** | 1.08 (1.06 - 1.10)*** |  |  |
| At least once a week | 1.33 (1.30 - 1.36)*** | 1.03 (1.02 - 1.05)*** | 1.18 (1.16 - 1.20)*** |  |  |
| Almost every day | 1.25 (1.16 - 1.34)*** | 0.93 (0.90 - 0.96)*** | 1.32 (1.27 - 1.36)*** |  |  |
| **Currently working** |  |  |  |  |  |
| No | Ref | Ref | Ref |  |  |
| Yes | 1.15 (1.13 - 1.17)*** | 0.91 (0.90 - 0.92)*** | 1.23 (1.21 - 1.24)*** |  |  |
| **Observations** | 269,171 | 248,620 | 269,171 |  |  |

**Note:** PR = Prevalence Ratio; 95% confidence interval (CI) in parentheses; Ref = Reference group.

*** p<0.001, ** p<0.01, * p<0.05
