# Supplementary material for: Association between male absence and women’s reproductive autonomy in sub-Saharan Africa: A cross-sectional analysis of Demographic and Health Survey Data
Source: PLOS Glob Public Health. 2026 Sep 15;6(9):e0007255. doi: 10.1371/journal.pgph.0007255 (PMC13577400; doi:10.1371/journal.pgph.0007255)
Supplement: Supplementary file 2 [file pgph.0007255.s002.docx]

**Table**

**S2 Appendix: Collinearity Diagnostics**

| **Variables** | **Variance Inflation Factor (VIF)** |
| --- | --- |
| Spousal Residence Status | 1.01 |
| Age | 1.56 |
| Education level | 1.54 |
| Wealth | 1.74 |
| Residence | 1.54 |
| Number of Children | 1.63 |
| Frequency of reading newspaper | 1.24 |
| Frequency of listening to radio | 1.20 |
| Frequency of watching television | 1.62 |
| Currently working | 1.05 |
| Country | 1.05 |
| **Mean VIF** | **1.38** |
